# Prebiotic Food Intake May Improve Bone Resorption in Japanese Female Athletes: A Pilot Study

**DOI:** 10.3390/sports9060082

**Published:** 2021-06-04

**Authors:** Tatsuya Ishizu, Eri Takai, Suguru Torii, Motoko Taguchi

**Affiliations:** 1 Graduate School of Sport Sciences, Waseda University, Saitama 359-1192, Japan; t.ishizu@toki.waseda.jp; 2 Waseda Institute of Sports Nutrition, Saitama 359-1192, Japan; eri.takai@jpnsport.go.jp (E.T.); shunto@waseda.jp (S.T.); 3 Sports Medical Center, Japan Institute of Sports Sciences, Tokyo 115-0056, Japan; 4 Faculty of Sport Sciences, Waseda University, Saitama 359-1192, Japan

**Keywords:** female athletes, prebiotics, gut microbiota, bone health, bone resorption

## Abstract

The aim of the present study was to clarify the influence of inulin and lactulose-fortified prebiotic food intakes on bone metabolism turnover among Japanese female athletes. The participants included 29 female athletes aged 18–25 years. They were requested to consume their habitual foods or drinks with one pack of prebiotic food every day for 12 weeks. Dietary intake, training time, body composition, blood sample, and fecal microbiota were assessed during this intervention period. Body composition, total energy intake, and training time of the participants revealed no significant changes during the intervention period. The occupation ratio of *Bifidobacterium* spp. was significantly increased at 3 and 4 weeks (18.0 ± 8.3% and 17.6 ± 8.5%, respectively) compared to that of pre-intervention (11.7 ± 7.3%) (*p* = 0.019 and *p* = 0.035, respectively). The serum TRACP-5b level was significantly decreased at 12 weeks (363 ± 112 mU/dL) compared to that at baseline (430 ± 154 mU/dL) (*p* = 0.018). These results suggest that the prebiotic food used in this study might have beneficial effects on bone health and gut microbial environment among female athletes. Further studies are warranted to identify the mechanism of the prebiotics–gut–bone axis.

## 1. Introduction

Inadequate energy intake can cause a variety of health problems in female athletes. The American College of Sports Medicine defined the concept of the Female Athlete Triad model in 1997 [1] and renewed it in 2007 [2]. This concept highlights the importance of adequate energy intake to assist the athletes’ health, such as bone health, reproductive function, and sport performance.

Poor bone health in female athletes occurs when athletes consume inadequate energy with or without disordered eating. Bone health can be assessed indirectly by assessing the bone metabolism turnover markers, which indicate bone formation or bone resorption [3], to provide useful information on dietary interventions [4,5,6]. For example, inadequate energy intake negatively affects bone metabolism turnover, leading to rapid increases in bone resorption and decreased bone formation [6,7]. Similarly, this modification contributes to reduced bone mineral density and increased risk of stress fractures [8,9]. Female athletes may be at an increased risk of bone disorders, including osteoporosis during or after retiring from their athletic careers [10]. Thus, adequate energy intake is essential for these athletes to prevent impaired bone health.

Nutritional intervention to improve bone health in female athletes addresses chronic energy deficiency and improves energy intake to meet the energy demands. A previous study reported that an increase in habitual energy intake is a useful nutritional strategy for solving women’s health problems [11]. However, many female athletes control their body weight on a daily basis because they believe that the lighter body weight, the better performance [12]. This idea is considered to cause energy intake deficiency. Nevertheless, it may be practically challenging to increase energy intake largely from the habitual diet for maintaining their body weight and sports performance. Calcium and vitamin D are also essential nutrients with a major role in bone health. Calcium is needed for bone mineralization, and vitamin D is needed for maintaining calcium homeostasis and bone remodeling, which have been suggested as protective against bone health among athletes [13]. However, the general incidence of low habitual dietary calcium and vitamin D intakes indicates [14] that some Japanese female athletes are likely to experience high bone metabolism turnover with a concomitant increase in the risk of bone disorders.

In recent years, the important function of prebiotics in the treatment of various diseases, including bone health, was studied. Inulin and lactulose are prebiotics, which are natural ingredients found in many foods. Inulin is generally found in root vegetables such as chrysanthemum, chicory, onion, garlic, and sweet potato [15], whereas lactulose is detected in heated milk [16]. Humans have consumed them for a long time. Inulin and lactulose have an energy content of 2.0 kcal per gram, which is half the calories of carbohydrates [17]. Prebiotics such as inulin and lactulose are selectively metabolized by microbes inside and on the body surface, and several studies have reported that both inulin and lactulose have prebiotic effects [18,19,20]. A prebiotic is defined as a substrate that is selectively utilized by host microorganisms, thus conferring health benefits [21]. These effects occur via prebiotic–microbial interactions in the large intestine. Moreover, a recent review summarized the gut microbiota involved in bone remodeling [22]. Furthermore, a previous study revealed that the combination of different types of prebiotics may have synergistic effects on health by allowing prolonged fermentation throughout the large intestine [23]. In animal studies, the combined intake of different types of prebiotics has shown to improve bone health [24,25]. Therefore, the combined intake of different types of prebiotics may provide nutritional benefits in female athletes. From these contexts, we have developed a new food with low energy including prebiotics such as inulin and lactulose. However, no studies have assessed whether the combination of different types of prebiotics affects bone health and gut microbiota among female athletes. Therefore, a better understanding of the combination effect of prebiotics among female athletes is essential.

The aim of the present study was to clarify the influence of inulin and lactulose-fortified prebiotic food intakes on bone metabolism turnover among Japanese female athletes.

## 2. Materials and Methods

### 2.1. Participants

Thirty female athletes aged 18–30 years were recruited in this study. One female athlete (long-distance running) was excluded due to personal reasons. In total, 29 of the 30 female athletes were intervened. The sport events were as follows: rhythmic gymnastics (*n* = 13), soccer (*n* = 7), middle- and long-distance running (*n* = 7) and figure skating (*n* = 2). Exclusion criteria were as follows: (1) regular use of medication that may influence metabolism or hormones; (2) injury; (3) smoking; (4) history of fractures in the last 6 months; (5) pregnancy; (6) milk allergy, and (7) use of oral contraceptives and antibiotics. All athletes were adequately informed about the study by verbal and written descriptions, and written informed consent was obtained before commencing the study. All procedures were approved by the Ethics Review Committee of Waseda University on Research with Human Subjects (approval number: 2017-096) following the Declaration of Helsinki. This study was registered in the University Hospital Medical Information Network Clinical Trials Registry (UMIN000029589).

### 2.2. Dietary Intervention

All participants were requested to take one pack of prebiotic food every day for 12 weeks. The participants were instructed to mix it with their daily diet according to their preference. This milk-flavored prebiotic food was manufactured by Morinaga Milk Industry Co., Ltd. (Tokyo, Japan) as per the request from the research team based on previous studies [26,27]. One pack (25 g) provided 100 kcal of energy, 3.5 g of protein, 2.8 g of fat, 15.0 g of carbohydrate, 2.5 g of inulin, 1.0 g of lactulose, 100 mg of calcium, and 0.5 µg of vitamin D. Prebiotic food was dispensed to each participant at baseline and on a regular basis thereafter. Participants were instructed to record their status of food intake during the intervention period. Moreover, these consumption records were cross-checked between each participant and research team. Empty packs and uneaten packs were collected from each participant during the regular food deliveries in order to monitor their compliance.

### 2.3. Anthropometric and Body Composition Measurements

Height was measured to 0.1 cm using a stadiometer (YG-200, Yagami, Aichi, Japan). Body weight (BW) was measured by the participants themselves to the nearest 0.05 kg using an electronic scale (UC-321, A&D, Tokyo, Japan) after urination and under morning fasting conditions during the intervention period. Body mass index (BMI) was calculated by dividing the body weight by height squared. Fat mass (FM), fat-free mass (FFM), bone area, bone mineral content (BMC), bone mineral density (BMD), and z-score of the whole body were measured using dual-energy X-ray absorptiometry (DXA) (QDR-4500DXA Scanner, Hologic; Marlborough, MA, USA) during the pre- and post-intervention periods. All scans were performed and analyzed using Hologic software (version 12.4.3, Hologic, MA, USA) by an orthopedic surgeon, who is one of the co-authors of this study.

### 2.4. Dietary Intake

The total energy intake (TEI) and macro- and micronutrient intake were investigated using a 3-day consecutive weighed food record with a food scale at pre-, mid-, and post-intervention periods. Participants were instructed to record all consumed food and beverages using the provided cooking scale (SD-004, Tanita Co., Ltd., Tokyo, Japan). Additionally, participants were also instructed to record their daily consumption and to take photos of all foods and beverages, including their labels. After completing the dietary record forms, the participants were interviewed by a well-trained dietitian, and their nutritional status was analyzed using the nutritional analysis software (version 2.85, Wellness 21, Top Business System, Okayama, Japan). Energy, macronutrient, and micronutrient intakes were calculated based on the Japanese Standard Food Composition Table 2015 published by the Ministry of Education, Culture, Sports, Science and Technology.

### 2.5. Training Time

Training time (hours/week) was assessed at pre- and post-intervention periods. All participants were requested to record their weekly training time to their questionnaires.

### 2.6. Analysis of Fecal Microbiota

Fecal microbiota samples were collected pre-intervention, at 1, 2, 3, 4, and 8 weeks, and post-intervention. These samples were collected by the participants themselves in collection tubes with guanidine thiocyanate solution (Techno Suruga Laboratory Co., Ltd., Shizuoka, Japan). The participants were trained to collect fecal collection before starting this intervention. In addition, the research team distributed stool collection cups to the participants to avoid contamination during the fecal collection. DNA was extracted from these samples, and the samples were analyzed by the terminal restriction fragment length polymorphism (T-RFLP) method. All fecal microbiota samples were analyzed using T-RFLP by Techno Suruga Laboratory Co., Ltd. (Shizuoka, Japan). The abundance of each terminal restriction fragment (T-RF) was calculated by first dividing them into 29 operational taxonomic units (OTUs) by the previous researches [28,29]. Each OTU was quantified as a percentage of the total OTU area, expressed as a percentage of the area under the curve (%AUC). The reference database, human fecal microbiota T-RFLP profiling (www.tecsrg.co.jp/t-rflp/t_rflp_hito_OTU.html, accessed on 3 June 2021) was used to putatively identify the bacteria in each classification unit and the corresponding OTU.

### 2.7. Blood Samples

Blood samples at pre-intervention, at 4 and 8 weeks, and post-intervention were collected in the morning after overnight fasting for bone metabolism turnover marker, bone-specific alkaline phosphatase (BAP), tartrate-resistant acid phosphatase 5b (TRACP-5b), 25-hydroxyvitamin D_3_ (25(OH)D_3_), and estradiol (E_2_). After clotting, the blood samples were separated via centrifugation at 3000 rpm for 15 min (Spectrafuge™ 6C, Labnet International Inc., Edison, NJ, USA), and the remaining serum was separated into aliquots and frozen at −80 °C until further analysis. TRACP-5b was analyzed using the enzyme immunoassay method. BAP was analyzed using an electro chemiluminescent immunoassay method. 25(OH)D_3_ was analyzed using the liquid chromatography–tandem mass spectrometry method. E_2_ was determined using a chemiluminescent immunoassay. All blood samples were analyzed by the LSI Medience Corporation (Tokyo, Japan).

### 2.8. Statistical Analyses

Data are presented as means ± standard deviations (SD) or medians with interquartile range. The Kolmogorov–Smirnov test was used to check the non-normal data. In order to achieve homogeneity, data were log transformed. Statistical analyses were performed using statistical software (SPSS ver. 25.0, IBM Corporation, Armonk, NY, USA). Repeated-measures one-way analysis of variance (ANOVA) was used to assess the differences in nutritional status, exercise status, fecal microbiota analysis, and blood sample analysis. Bonferroni post hoc corrections for equal variance and the Games–Howell procedure for unequal variance were used to identify significantly different measurement points. The paired *t*-test or Mann–Whitney test were used to compare between the physical characteristics data during the pre- and post-intervention period. Statistical significance was set at *p* < 0.05 in two-sided tests for all analyses.

## 3. Results

### 3.1. Participants Characteristics

The intake rate of prebiotic food during intervention was 76 ± 21%. The comparison of body composition and bone parameter variables at pre- and post-intervention is presented in Table 1. No significant changes in any parameter, including body weight, were observed after the intervention.

### 3.2. Dietary Status

The energy, and macro- and micronutrient intakes of the participants during intervention are presented in Table 2. TEI, protein, fat, carbohydrate, calcium, vitamin D, vitamin K and dietary fiber intakes at mid- and post-intervention were not significantly different compared with that at pre-intervention. Only iron intake was significantly different in this intervention.

### 3.3. Fecal Microbiota Analyses

**Figure 1:**
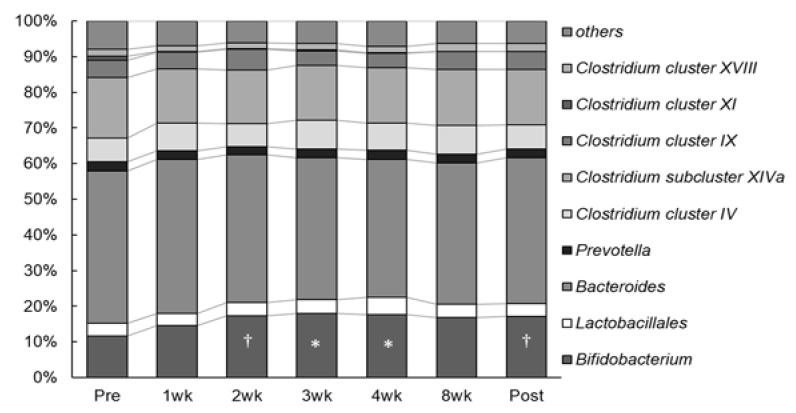
Change in gut microbiota composition at 12 weeks. * *p* < 0.05 versus pre-intervention at the same genus. † *p* < 0.10 versus pre-intervention at the same genus. No significant differences were found in the gut microbiota without *Bifidobacterium* spp.

Human gut microbiota composition mainly comprises members of approximately 10 bacterial flora groups (Figure 1). As illustrated in Figure 1, one of the fecal microbiotas significantly changed during the intervention. The occupation ratio of *Bifidobacterium* spp. was significantly increased at 3 and 4 weeks (18.0 ± 8.3% and 17.6 ± 8.5%, respectively) compared to that during pre-intervention (11.7 ± 7.3%) (*p* = 0.019 and *p* = 0.035, respectively), and further, an increasing trend was observed at 2 week and post-intervention (17.3 ± 7.0% and 17.1 ± 7.6%, respectively) compared to pre-intervention (*p* = 0.057 and *p* = 0.073, respectively). No statistically significant changes were observed in the composition of other fecal microbiota during the intervention.

### 3.4. Changes in Bone Markers

**Figure 2:**
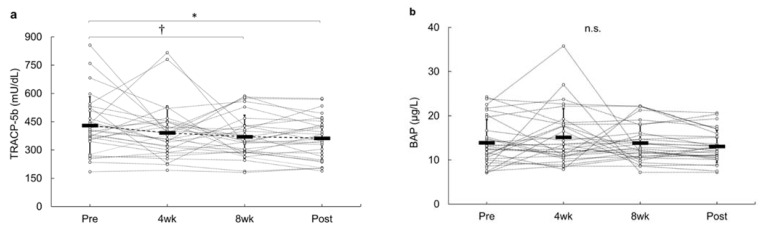
Change from pre-intervention for bone-related markers during 12 weeks of the intervention. (**a**) Change in TRACP-5b for 12 weeks. (**b**) Change in BAP for 12 weeks. * *p* < 0.05 was considered as statistically significant. † *p* < 0.10 was considered as trend toward a significant.

Figure 2a,b depict the changes in bone metabolism turnover markers at pre-intervention, weekly, and post-intervention periods. The serum TRACP-5b level decreased significantly at 8 weeks and post-intervention in this study. Post hoc testing revealed that TRACP-5b at post-intervention (363 ± 112 mU/dL) was lower than that at pre-intervention (430 ± 154 mU/dL) (*p* = 0.018), and TRACP-5b at 8 weeks (370 ± 114 mU/dL) revealed a decreasing trend compared to that with the pre-intervention (*p* = 0.059). No significant changes were observed in BAP during the intervention. E_2_ and 25(OH)D_3_ levels from pre- to post-intervention did not reveal any significant change during the intervention.

## 4. Discussion

The main finding of this study revealed that inulin and lactulose-fortified prebiotic food intakes for 12 weeks suppressed bone resorption marker and increased the occupation ratio of *Bifidobacterium* spp. in the gut microbiota without body weight gain among the Japanese female athletes. This finding provides novel insights into the existing literature, indicating that the combined intake of different types of prebiotics may play a pivotal role in improving bone health among Japanese female athletes.

Inulin is an indigestible, water-soluble dietary fiber, that is, a polysaccharide with a glucose molecule attached to the reducing end of the fructose chain [30], whereas lactulose is a synthetic disaccharide comprising fructose and galactose that cannot be digested or absorbed by humans [16]. These prebiotics are functional food ingredients and have been used in human studies. Previous studies have reported that the short-term administration of inulin or lactulose increased the occupation ratio of *Bifidobacterium* spp. in the gut microbiota [18,31,32], which is responsible for the growth of health-promoting bacterial species. Those observations are in agreement with the finding of our study (Figure 1). According to a consensus statement [21], prebiotics were recognized to particularly stimulate *Bifidobacterium* spp. (bifidogenesis). Thus, the combination of inulin and lactulose in this study might contribute to the “bifidogenic effect”. Moreover, the administration of inulin or lactulose reduced bone resorption markers in postmenopausal women and healthy young men [33,34,35]; however, only limited studies have demonstrated that supplementation with these prebiotics change the gut microbiota and bone metabolism turnover markers in humans [35]. In our study, the changes in bone metabolism turnover were identified after the changes *Bifidobacterium* spp. in the gut microbiota. A recent review summarized that prebiotics may indirectly or directly manipulate the gut–bone axis to improve bone health [22]. Increasing the occupation ratio of *Bifidobacterium* spp., short-chain fatty acids (SCFAs) levels [35], and suppression of pro-inflammatory cytokines [36] are possible pathways for the microbial manipulations to the improvement of bone health. Prebiotic intakes affected the gut microbiota and promoted SCFAs production [37]. In particular, *Bifidobacterium* species stimulate SCFAs productions by the intestinal microbiome [38], and associated with an increased production of SCFAs [39]. SCFAs are metabolites generated by gut microbial fermentation from prebiotics [40]. Acetic acid, butyric acid, and propionic acid are the main SCFAs produced by microorganisms in the intestine [41]. A previous study reported that SCFAs regulate the direct suppression of osteoclast synthesis and decrease bone resorption [42]. Mechanistically, this is thought to be because propionic acid and butyric acid inhibit osteoclast signal transduction substances and prevent osteoclast differentiation. After ingesting prebiotic food for 2 weeks, the occupation of *Bifidobacterium* spp. was higher than that before intervention and was maintained at a higher level until post-intervention in this study. Therefore, it is inferred that increases in the *Bifidobacterium* spp. induced the inhibition of bone resorption in this study as well.

Energy intake deficiency may exhibit uncoupled bone metabolism turnover, which decreases bone formation, increases bone resorption and/or a combination of the two, thereby consequently inducing unfavorable bone disorders. Subsequent reduction in the estrogen levels and associated reproductive dysfunction may also be indirectly influenced by bone health among the female athletes. Since serum E_2_ plays a crucial role in maintaining normal bone health in women, De Souza et al. [43] reported that low E_2_ levels were involved with suppressed bone formation and increased bone resorption. Since E_2_ did not show any significant changes during this intervention, it is inferred that E_2_ did not affect bone metabolism turnover. Participants in this study were in an energy intake deficient state, and the bone resorption marker during their pre-intervention was promoted. Indeed, the value of bone resorption marker was above the reference value [44]. These results were in accordance to those observed in previous studies [7,45]. In particular, energy intake deficiency elevates bone resorption markers in athletes. If the balance between bone resorption and bone formation is maintained (coupling), bone mass and bone mineral contents remain balanced; however, when uncoupling in bone metabolism turnover occurs and bone resorption becomes more dominant, bone mineral density decreases. TRACP-5b is recognized as a bone resorption marker with low intra- and inter-day variations [46]. Furthermore, this marker is a predictor of the risk of stress fractures in female athletes [47]. In this study, participants who had elevated bone resorption markers before the intervention had a positive effect from consuming the prebiotic foods (Figure 2a). The finding of reduced bone resorption marker in this study may prove beneficial for preventing issues related to bone health among female athletes.

As well as energy intake, key nutrients for bone health such as calcium, vitamin D and vitamin K intakes is also important in athletes. The adequate intake of these nutrients is one of the essential nutritional strategies that female athletes can utilize to reduce the risk of bone injuries [48]. Regarding these nutrient intakes, participants did not change their dietary intake throughout the study. However, calcium, vitamin D, and vitamin K intakes are lower than the dietary reference intakes (DRIs) in Japan. The previous studies suggested that an increase in certain micronutrients (i.e., calcium, vitamin D and vitamin K) has a favorable effect on bone health [49,50]. Although there was no significant change in these nutrient intakes during this intervention period, it should be noted that these nutrients were low. Since low intakes of these nutrients are a characteristic of the dietary habits among Japanese female athletes [14], there is a need to develop a nutritional approach to improve this problem. Iron intake also differed significantly during the intervention period in this study. Although iron is an essential micronutrient for humans, large amounts of iron intakes might cause gut inflammation, which might be due to irritation of the gut mucosa, adverse alterations in the gut microbiota, or both [51]. However, the significant changes in our study were within the DRIs in Japan and did not have adverse effects on the gut microbiota.

Body composition is related to athletic performance [52,53,54]. During the intervention, no changes were observed in BW, FFM, and FM. Cialdella-Kam et al. [55] found that a 6-month intervention provided extra energy (+360 kcal/day) that could successfully reverse the menstrual status, resulting in weight gain (+1.6 kg). In contrast, our study did not induce substantial weight gain (+0.1 kg). While habitual EI increment and body weight gain are useful strategies for solving women’s health problems [11,56], such strategies may not be practically feasible for long-distance runners and rhythmic gymnasts. Increased body fat reduces athletic performance [57]. FM did not change significantly in our study. Among the competing female athletes that require daily weight control, the prebiotic food used in this study may provide a “bone protection effect” without having a significant impact on body composition.

The advantage of this study is that it was conducted in multiple events where female athletes participated in intense training every day. Previous studies were conducted in postmenopausal women and adolescent girls. The rate of bone turnover varies with age; therefore, it was unclear whether prebiotic intake in female athletes would prove beneficial for bone health. Thus, the present findings can be used as a nutritional strategy in sports fields. Future athletes’ studies using probiotics or synbiotics are needed, as probiotic intake has reported to have a protective effect on bone parameter in rats [58]. In contrast, this study has certain limitations. First, our participants could not perform placebo trials. As our participants in this study had a high-performance level, it was difficult to get such athletes to commit to a control trial such as a long-term crossover intervention. Second, we did not measure SCFAs in fecal samples or serum proinflammatory cytokines. Thus, our study could not explicitly explain the prebiotics–gut–bone axis. Future studies need to re-evaluate the impact of prebiotic food on gut microbiota and bone metabolism turnover in other athletes.

In conclusion, intake of inulin and lactulose-fortified prebiotic food over 12 weeks might suppress bone resorption marker and increase the occupation ratio of *Bifidobacterium* spp. in feces without body weight gain among Japanese female athletes. The findings of this study can be applied to prevent bone health-related disorders among female athletes.

## Figures and Tables

**Table 1:** Physical characteristics of the participants during the interventions.

	Pre	Post	*p*-Value
Age (y)	20 ± 1	-	-
Height (cm)	160.2 ± 5.3	-	-
Body weight (kg)	51.2 ± 5.5	51.3 ± 5.3	0.620
BMI (kg/m^2^)	19.9 ± 1.7	20.0 ± 1.8	0.557
Fat mass (kg)	9.8 (7.0, 10.6)	9.3 (7.4, 10.9)	0.184
Fat-free mass (kg)	40.6 ± 4.2	40.7 ± 4.4	0.902
Bone area (cm^2^)	1845 ± 118	1842 ± 125	0.620
BMC (g)	2109 ± 189	2096 ± 215	0.557
BMD (g/cm^2^)	1.143 ± 0.069	1.136 ± 0.066	0.184
Whole body z-score	1.3 ± 1.2	1.2 ± 1.2	0.242
Training time (hours/week)	25.6 ± 10.0	27.3 ± 13.5	0.474

Data were expressed as mean ± SD or median (inter quartile range); BMI, body mass index; BMC, bone mineral contents; BMD, bone mineral density. *p* values < 0.05 represent significantly different mean.

**Table 2:** Energy, macro, and micro-nutrient intakes of the participants.

	Pre	Mid	Post	*p*-Value
TEI (kcal)	1724 ± 441	1672 ± 524	1603 ± 575	0.295
Protein (g)	63.7 ± 24.1	65.8 ± 28.5	63.8 ± 30.4	0.774
Fat (g)	59.1 ± 18.1	60.8 ± 22.1	57.3 ± 26.2	0.688
Carbohydrate (g)	228.4 ± 62.0	209.2 ± 66.8	203.4 ± 67.1	0.069
Calcium (mg) ^a^	418 (316, 713)	480 (290, 765)	364 (231, 694)	0.088
Iron (mg) ^a^	5.4 (3.7, 10.3)	10.6 (5.9, 13.1)	5.2 (3.6, 10.4)	<0.001
Vitamin D (μg) ^a^	4.4 (2.4, 7.9)	3.3 (2.3, 7.6)	3.6 (1.7, 9.6)	0.998
Vitamin K (μg) ^a^	146 (68, 326)	127 (58, 382)	171 (68, 314)	0.787
Dietary fiber (g)	11.5 ± 6.6	12.7 ± 6.0	10.7 ± 6.3	0.086

Data were expressed as mean ± SD or median (inter quartile range). TEI, total energy intake. ^a^ Log transformed variables for non-normally distributed variables were used for analysis. Mid date revealed the nutritional composition of prebiotic food intake.

## Data Availability

The datasets generated and/or analyzed during this study are not publicly available because our ethical approval did not include the use of these data by other researchers.
